# Supplementary material for: Measuring multi-dimensional disparity index: A case of Nepal
Source: PLoS One. 2023 Oct 5;18(10):e0286216. doi: 10.1371/journal.pone.0286216 (PMC10553211; doi:10.1371/journal.pone.0286216)
Supplement: S1 Appendix — (DOCX) [file pone.0286216.s001.docx]

# Appendix

**Figure S1.** Political map of Nepal and its provinces.


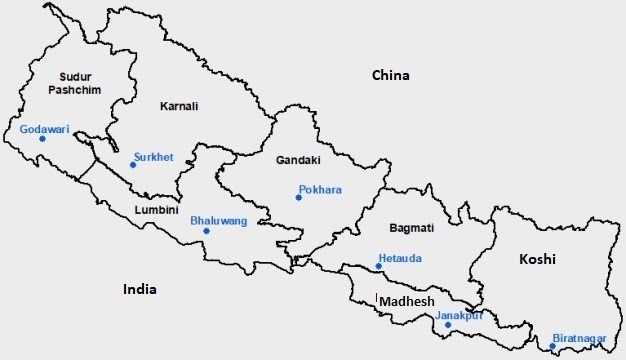


*Source: National Statistics Office (NSO), 2022

**Table S1.** Brief information on provinces in Nepal.

| Province | Location | Geography | Population | Areas (Km square) | No. of local governments |
| --- | --- | --- | --- | --- | --- |
| Koshi | East | Mountain, Hill, and Tarai | 4,534,943 | 25,905 | 137 |
| Madhesh | Southern east | Tarai | 5,404,145 | 9,661 | 136 |
| Bagmati | Middle east | Mountain, Hill, and Tarai | 5,529,452 | 20300 | 119 |
| Gandaki | Middle west | Mountain, Hill, and Tarai | 2,403,757 | 21504 | 85 |
| Lumbini | Southern west | Mountain, Hill, and Tarai | 4,499,272 | 22,288 | 109 |
| Karnali | Northern west | Mountain and Hill | 1,570,418 | 27984 | 79 |
| Sudur Pashchim | Far west | Mountain, Hill, and Tarai | 2,552,517 | 19,915 | 88 |

**Table S2**. Sources of data.

| **S.No.** | **Sources** | **Contributed Dimensions of MDI** | **Availability** |
| --- | --- | --- | --- |
| 1 | NPC & Central Bureau of Statistics (CBS, 2020) | Economy | Availability on the request to the CBS  https://cbs.gov.np/ |
| 2 | CBS (2022) | Economy, Health, Education, Living Standards, Demography | Partially available on <http://nationaldata.gov.np/>  and other data will be available on request |
| 3 | Ministry of Federal Affairs and General Administration (MOFAGA, 2019); | Economy, Geography and climatic vulnerabilities | Availability on the request to the MOFAGA  https://www.mofaga.gov.np/ |
| 4 | Department of Forest Research and Survey (DOFRS, 2018) | Economy | https://frtc.gov.np/downloadfile/Forests%20Cover%20Maps%20of%20Local%20Levels%20in%20Nepal%20Summary%20(1)_1568111767(2)_1572858696.pdf |
| 5 | Department of Health Services (DOHS, 2019) | Health | https://dohs.gov.np/ihims-raw-data/ |
| 6 | Centre for Education and Human Resource Development (CEHRD, 2021) | Education | https://www.cehrd.gov.np/file_data/mediacenter_files/media_file-17-98334697.pdf |
| 7 | CBS (2019) | Geography and climatic vulnerabilities | Partially available on <http://nationaldata.gov.np/>  and other data will be available on request |
| 8 | Department of Hydrology and Meteorology (DOHM, 2017) | Geography and climatic vulnerabilities | https://opendatanepal.com/dataset/observed-climate-trend-analysis-of-nepal-1971-2014 |
| 9 | Nepal Disaster Risk Reduction Portal (NDRRP, 2022) | Geography and climatic vulnerabilities | http://www.drrportal.gov.np/ |

*Some government authorities do not publish data on the website but make it public in print copies. The data are available upon request.
