# Supplementary material for: Measuring multi-dimensional disparity index: A case of Nepal
Source: PLoS One. 2023 Oct 5;18(10):e0286216. doi: 10.1371/journal.pone.0286216 (PMC10553211; doi:10.1371/journal.pone.0286216)
Supplement: Attachment — Submitted filename: Response to Reviewers.docx [file pone.0286216.s002.docx]

# Dear editor and reviewers

Plos One.

Thank you for insightful review comments and of course they were very helpful to improve the quality of paper. In the following table, we mention our responses to the reviewers’ comments.

| **Commented By** | **Comments** | **Remarks** |
| --- | --- | --- |
| Editor | If there are ethical or legal restrictions on sharing a de-identified data set, please explain them in detail (e.g., data contain potentially sensitive information, data are owned by a third-party organization, etc.) and who has imposed them (e.g., an ethics committee). Please also provide contact information for a data access committee, ethics committee, or other institutional body to which data requests may be sent. | The data we used in this study are publicly available in the respective agencies’ report. In the manuscript, we add a table mentioning the source of data. It is in the supplementary file. |
|  | If there are no restrictions, please upload the minimal anonymized data set necessary to replicate your study findings as either Supporting Information files or to a stable, public repository and provide us with the relevant URLs, DOIs, or accession numbers. For a list of acceptable repositories, please see <http://journals.plos.org/plosone/s/data-availability#loc-recommended-repositories>.  We will update your Data Availability statement on your behalf to reflect the information you provide. | We added sources of data. These data basically were made public in the websites of respective government authorities. However, we took a consent from each of the authority for using the data.  However, some of the data were not published in the web page. We received them in print version. In this case, we refer to the concerned authorities’ web page and data can be available upon the request. (please see supplementary file) |
|  | Please include your tables as part of your main manuscript and remove the individual files. Please note that supplementary tables (should remain/ be uploaded) as separate ""supporting information"" files. | Adjusted in Table 1- 5 and supporting information in separate file in the Annex. In the supporting information, we added political map of Nepal, brief information on the provinces of Nepal, and data availability statements. |
|  | Abstract should be written in concise. I would suggest listing only some of the most important results to justify the implications and conclusions of the study. | We revisit the abstract hopefully this satisfies the reviewers. |
|  | The background of an introduction should be revised accordingly. The introduction is very good. It doesn't reflect the goal; please rewrite it again, it is suggested to include some latest reference. Objectives of this study must be included at end of introduction part. | We incorporate purposes of this study at the end of introduction section as writing “intends to assess the Multi-dimensional Disparity Index of Nepal in all seven provinces. …….”  We also include latest relevant references like Lewison et al. (2020); Poudel et al. (2020); CEHRD (2022) and many more. (Please see pp. 1-4) |
|  | I highly recommended to authors, if possible, please modify the figure with good quality images. | Adjusted Table 1-5 and also in supplementary table in Annex |
|  | The economic intuition behind the results are missing. | The purpose of the study is to develop MDI and replicate it to assess the multiple disparities in a country. We added purpose of the study in the introduction section and discuss the result in shade of other research result to make clear the intuition behind the result. |
|  | The author/s should revise the discussion part. The result should be supported with recent studies. | We revise it and also add recent literature as suggested (please see p. 17-19 |
|  | What is contribution of this work to existing literature? | We add how multidimensional disparity index provides a wholistic approach in measuring multidimensional disparity which also different from existing mono dimensional approach. Added in introduction and in the conclusion section as well. “study provides a robust approach to measuring disparity in multiple dimensions…..” |
|  | It has been observed that the authors have used old references and ignored the latest studies. So, it is suggested to add recent references. Please check reference section some references are missing. | Adjusted in the reference section and we also incorporated new references like Lewison et al. (2020); Poudel et al. (2020); CEHRD (2022) and many more |
|  | The policy implications also required elaboration. The implications should go along with the results and the course of action should be discussed in this part. | Adjusted in the conclusion section. P. 21. We add the implications of the study in conclusion section for policy maker that the study will support for executing inclusive governance and to the future researcher a robust approach ….. |
|  | In some places, some grammatical errors are found that need to be fixed. | We edited language of the manuscript. |
| Reviewer I | What are the limitations of the MDI index, and what are the advantages of the index for Nepal? Are there any limitations for Nepal? λIn the introduction, the purpose of the study and the significance of the study should be further clarified. | Limitations and advantages of index are adjusted in the conclusion section from p. 21 as unit of analysis as local governments etc.  Introduction, purpose, and significance were further clarified in the introduction section, in p. 1-4. |
|  | The number of government officials, international NGO workers, MDI experts, economists, academicians, announcement health experts and sociologists involved in determining MDI dimensions and indicators in the research methodology. And their reliability and validity in this process, such as t-test. | Adjusted in methodology section. We add validity as a separate subsection at the end of methodology section. |
|  | It is suggested that the basic profiles of the seven provincial governments of Nepal, which are the subject of the study, should be introduced in the research methodology including physical geography, climate, economy, culture, population, politics, education, etc. | Adjusted in methodology section as a separate new subsection as "Basic descriptions about subject of this study" |
|  | The results are only a relatively simple description of Table 2, without deeper excavation, not to mention the analysis of the actual situation in the provinces and cities. | We added “Distribution of Local Governments across its MDI values within Provinces” as a separate sub-section in this result section. |
|  | The discussion does not provide a good argument for the implementability of MDI index in Nepalese applications and its scientific validity. | The study propose was to assess the disparity status of at different level of governments. The discussion section provides the analysis on disparity across six dimensions at provincial as well as national level. This will have an important policy implication for policymakers making equity based resource distribution rather than equality. We added this concern in text. |
|  | The discussion should further analyze and draw out the particularities and commonalities of Nepal and its provinces in terms of economy, education, health, geography, population, and life, and the reasons for these individualities and commonalities. | We added comparison of the index among/between the provinces. (Please see discussion section) |
| Reviewer II | Research title may be revised. | Adjusted with revision in the title |
|  | Most of the references / citations mentioned in the article are very old | We included new references like Lewison et al. (2020); Poudel et al. (2020); CEHRD (2022) and many more |
|  | the research objectives and rationale required to be properly specified. | We specifically revisited the introduction making it clearer. The purpose of this study is included at the end of introduction section as writing “intends to assess the Multi-dimensional Disparity Index of Nepal in all seven provinces. …….” |
|  | Research design is weak. | We enrich research design by adding information about subject, sources of data, and validity section and many more in the methodological parts. |
|  | Article proofreading is highly recommended. | Language editing was done. |
|  | The study period to calculate MDI not mentioned to assess or make conclusions. | We included the time frame of collected data. The data in the study covers 2017-2022. (see Supplementary file, Annex III) |
